# SRSF1 regulates exosome microRNA enrichment in human cancer cells

**DOI:** 10.1186/s12964-020-00615-9

**Published:** 2020-08-20

**Authors:** Yi-Fan Xu, Xiaohui Xu, Amy Gin, Jean D. Nshimiyimana, Blaine H. M. Mooers, Massimo Caputi, Bethany N. Hannafon, Wei-Qun Ding

**Affiliations:** 1 Department of Pathology, University of Oklahoma Health Sciences Center, Oklahoma City, 940 Stanton L. Young Blvd., BMSB 401A, Oklahoma City, OK 73104 USA; 2 Department of General Surgery, First People’s Hospital of Taicang City, Taicang Affiliated Hospital of Soochow University, Suzhou, 215400 China; 3 Department of Biochemistry and Molecular Biology, University of Oklahoma Health Sciences Center, Oklahoma City, OK 73104 USA; 4 Charles E. Schmidt College of Medicine, Florida Atlantic University, Boca Raton, FL 33431 USA; 5 Department of Obstetrics and Gynecology, Section of Gynecologic Oncology, Stephenson Cancer Center, College of Medicine, University of Oklahoma Health Sciences Center, Oklahoma City, OK 73103 USA; 6 Stephenson Cancer Center, University of Oklahoma Health Sciences Center, Oklahoma City, OK 73104 USA

**Keywords:** SRSF1, Exosome, miRNA, miR-1246, Pancreatic cancer

## Abstract

**Background:**

Exosomes are extracellular vesicles containing a variety of biological molecules including microRNAs (miRNAs). We have recently demonstrated that certain miRNA species are selectively and highly enriched in pancreatic cancer exosomes with miR-1246 being the most abundant. Exosome miRNAs have been shown to mediate intercellular communication in the tumor microenvironment and promote cancer progression. Therefore, understanding how exosomes selectively enrich specific miRNAs to initiate exosome miRNA signaling in cancer cells is critical to advancing cancer exosome biology.

**Results:**

The aim of this study was to identify RNA binding proteins responsible for selective enrichment of exosome miRNAs in cancer cells. A biotin-labeled miR-1246 probe was used to capture RNA binding proteins (RBPs) from PANC-1 cells. Among the RBPs identified through proteomic analysis, SRSF1, EIF3B and TIA1 were highly associated with the miR-1246 probe. RNA immunoprecipitation (RIP) and electrophoretic mobility shift assay (EMSA) confirmed the binding of SRSF1 to miR-1246. Lentivirus shRNA knockdown of SRSF1 in pancreatic cancer cells selectively reduced exosome miRNA enrichment whereas GFP-SRSF1 overexpression enhanced the enrichment as analyzed by next generation small RNA sequencing and qRT-PCR. miRNA sequence motif analysis identified a common motif shared by 36/45 of SRSF1-associated exosome miRNAs. EMSA confirmed that shared motif decoys inhibit the binding of SRSF1 to the miR-1246 sequence.

**Conclusions:**

We conclude that SRSF1 mediates selective exosome miRNA enrichment in pancreatic cancer cells by binding to a commonly shared miRNA sequence motif.

**Video Abstract**

**Graphical abstract:**

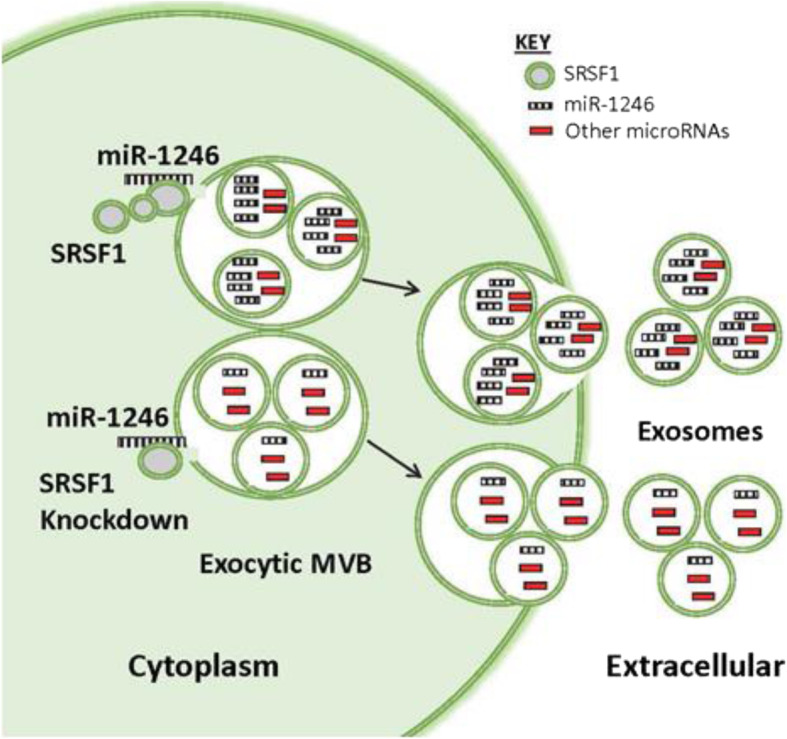

## Background

Exosomes are endosome-derived extracellular vesicles (EVs) [1] that can be transferred from cancer cells to stromal cells in the tumor microenvironment [2, 3]. These membrane vesicles are 40–120 nm in size, and contain proteins, lipids and nucleic acids, including small RNAs such as microRNAs (miRNAs) [1, 4]. Exosome-mediated intercellular communication between cancer cells, endothelial cells [5, 6], fibroblasts [7, 8], or immune cells [9, 10] can facilitate tumor progression. Furthermore, cancer exosomes are released into the circulation and contribute to pre-metastatic niche formation in distant organs [11, 12].

How cancer exosomes interact with stromal cells to promote tumor progression has been extensively investigated. One critical signaling event in the tumor microenvironment is the exosome miRNA-mediated intercellular communication [1, 13–15]. Studies have shown that exosome miRNA signaling promotes tumor progression in various model systems [16, 17]. Notably, it has been reported that miRNAs contained in exosomes are delivered to recipient cells in the tumor microenvironment or distant organs where they can regulate target gene expression and promote tumor angiogenesis and metastasis [13, 14, 18].

In the context of exosome miRNA signaling, we and others have reported that certain miRNA species are selectively enriched in cancer exosomes as compared to exosomes derived from normal epithelial cells [13, 19–21]. Results from several studies have also indicated that selective enrichment of exosome miRNAs is relevant to tumor progression [22]. For example, exosome sorting of miR-193a was found to promote colon cancer progression [23]. Likewise, miR-122, a cancer exosome enriched miRNA [19, 24], was shown to reprogram glucose metabolism in a pre-metastatic niche to facilitate metastasis in a breast cancer model system [25]. Moreover, the exosome enriched miR-1246 [26] was reported to promote tumor invasion in both breast cancer [27] and oral squamous cell carcinoma [28]. It seems clear that selective enrichment of exosome miRNAs drives cancer exosome miRNA signaling in the tumor microenvironment, which in turn reinforces tumor invasiveness and progression. However, how exosome miRNAs are enriched or how exosome miRNA signaling is initiated in cancer cells remains largely unknown. Elucidating the mechanisms of selective exosome miRNA enrichment in cancer cells may help identify new cancer therapeutic opportunities that are urgently needed.

Recent reports have indicated that certain RNA binding proteins (RBPs) are involved in exosome miRNA sorting in eukaryotic cells, and the type of RBPs involved seems to differ among various model systems [23, 29, 30], suggesting that exosome miRNA sorting is a tissue or cell type specific process. Furthermore, there have been no reports on the identification of RBPs that regulate exosome miRNA sorting in pancreatic cancer cells. We have recently characterized the biogenesis of exosome miR-1246 [26], which is the most highly enriched miRNA in pancreatic cancer cell-derived exosomes [21]. The aim of this study was to utilize our established cell model systems to identify RBPs that are involved in exosome miRNA loading in pancreatic cancer cells. Using a labeled miR-1246 probe as “bait”, we fished out several RBPs from pancreatic cancer cells, including serine and arginine rich splicing factor 1 (SRSF1), eukaryotic translation initiation factor 3 subunit B (eIF3B), and T cell-restricted intracellular antigen 1 (TIA1). We found that SRSF1, a recently claimed oncoprotein [31], is predominantly involved in regulating exosome miRNA enrichment in pancreatic cancer model systems.

## Methods

### Cell culture

The human pancreatic cancer cell lines PANC-1, MIAPaCa-2 and BxPC-3, and breast cancer cell line MDA-MB-231 were obtained from the American Type Culture Collection (ATCC, Manassas, VA, USA). Cells were cultured following ATCC’s instructions except that exosome-depleted fetal bovine serum (FBS) and horse serum were applied whenever needed. Exosome-depleted FBS and horse serum were prepared by pelleting the serum exosomes at 200,000×g for 2 h at 4 °C. Cells were routinely incubated in a humidified environment at 37 °C and 5% CO_2_.

### Exosome isolation

**Fig. 1:**
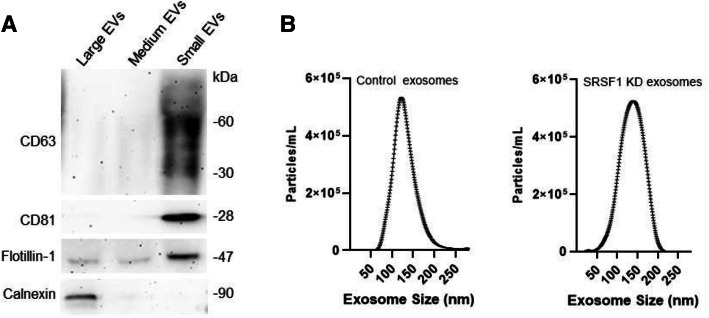
Verification of the exosomes derived from PANC-1 cells. **a** Representative western blot analysis of CD63 (non-reducing condition), CD81, flotillin, and calnexin in the EVs isolated from PANC-1 cells. Positive exosome markers are only detected in small EVs (exosomes). **b** Representative nanoparticle tracking analysis of exosomes (small EVs) derived from control and SRSF1 knockdown PANC-1 cells. Three individual experiments were performed for both **a** and **b**

Exosomes were isolated from the culture medium utilizing a combination of centrifugation, ultracentrifugation, and filtration as we recently described [13, 19, 26], with minor modifications. In brief, the culture medium of PANC-1 cells was pre-cleared by 10,000 g centrifugation for 30 min at 4 °C, and the resulting supernatant was filtered through a 0.22 μm PVDF centrifuge filter. The large size EVs were trapped in the filter and recovered in PBS. The filtered supernatant was then applied to a 0.1 μm PVDF centrifuge filter. The medium size EVs were trapped in the second filter and re-suspended in PBS. The small size EVs (exosomes) in the final supernatant were recovered by ultracentrifugation (100,000 g, 70 min at 4 °C). The isolated exosomes were verified by western blot detecting positive and negative exosome marker proteins and nanoparticle analysis (Nanosight NS300 System, Malvern Instruments, UK) measuring both sizes and concentrations of the isolated exosomes (Fig. 1).

### miRNA binding protein pull-down

Pull-down experiment was performed using the Pierce™ Magnetic RNA-Protein Pull-Down Kit (Thermo Fisher Scientific). Briefly, 100 pmol of biotin-labeled miR-1246 or poly-A RNA oligonucleotides (Integrated DNA Technologies) were hybridized to 100 μl streptavidin magnetic beads (Prod#1862766, Thermo Fisher Scientific). The miR-1246-biotin-streptavidin beads were incubated with PANC-1 lysate for 60 min at 4 °C. The lysate-bead mixture was washed three times with washing buffer from the above-mentioned kit. To elute bound proteins, 50 μl of elution buffer was applied, and a magnetic separator was applied to separate the beads from the eluted protein, following the manufacturer’s protocol (Pierce™ Magnetic RNA-Protein Pull-Down Kit, Thermo Fisher scientific). Proteins were separated by SDS-PAGE before mass spectrometry (MS) analysis.

### Liquid chromatography–mass spectrometry (LC-MS)/mass spectrometry (MS) measurement

The experiment was performed by the Laboratory for Molecular Biology and Cytometry Research Core Facility at OUHSC. Proteins were digested with trypsin according to the FASP [32] protocol. Briefly the eluate was buffer exchanged in 8 M urea, the proteins were reduced with 10 mM dithiothreitol and then alkylated with 10 mM iodoacetamide. The peptides were eluted, dried and resuspended. Liquid chromatography tandem mass spectrometry was performed by coupling a nanaoAcquity UPLC (Waters Corp., Manchester, UK) to a Q-TOF SYNAPT G2S instrument (Waters Corp., Manchester, UK). Each protein digest (about 100 ng of peptide) was delivered to a trap column (300 μm × 50 mm nanoAcquity UPLC NanoEase Column 5 μm BEH C18, Waters Corp, Manchester, UK) at a flow rate of 2 μl/min in 99.9% solvent A (10 mM ammonium formate pH 10, in HPLC grade water). Tandem mass spectra were generated in the trapping region of the ion mobility cell by using a collisional energy ramp from 20 V (low mass, start/end) to 35 V (high mass, start/end). The pusher/ion mobility synchronization for the HDMSe method was performed using MassLynx V4.1 and DriftScope v2.4. LockSpray of Glufibrinopeptide-B (m/z 785.8427) was acquired every 60 s and lock mass correction was applied post acquisition.

### Protein identification

Raw MS data were processed by PLGS (ProteinLynx Global Server, Waters Corp., Manchester, UK) for peptide and protein identification. MS/MS spectra were searched against the Uniprot Human database (containing 20,417 reviewed sequences) with the following search parameters: full tryptic specificity, up to two missed cleavage sites; carbamidomethylation of cysteine residues was set as a fixed modification; and N-terminal protein acetylation and methionine oxidation were set as variable modifications.

### Small RNA library preparation and next generation sequencing

Total RNA was extracted from cell and exosome pellets using the TRIzol reagent (Invitrogen/Life Technologies, Carlsbad, California). The small RNA libraries were constructed and run on the Illumina MiSeq platform as we recently described [21, 26].

### RNA immunoprecipitation assay

PANC-1 cells or MDA-MB-231 cell lysates were prepared using IP buffer (10 mM Tris-HCl, pH 7.4, 50 mM NaCl, 0.5 mM EDTA, 1 mM PMSF, and 1% Triton X-100). The lysate was sonicated for 1 min on ice, and insoluble material was removed by centrifugation. Supernatants were collected, and protein concentrations were measured. The supernatant was pre-cleared by Protein G Dynabeads™(Thermo Fisher Scientific), and then mixed with antibody: SRSF1 (Santa Cruz, sc-33,652), EIF3B (Santa Cruz, sc-137,214), TIA1 (Santa Cruz, sc-166,247), GAPDH (ProMab, 20,035), and IgG (Santa Cruz sc-2025) in a ratio of 1:100 at 4 °C overnight with gentle rotation. To capture the antibody-protein-RNA complexes, 50 μl of protein G magnetic beads were added, and the complexes were rotated for 2 h at 4 °C. The sample was separated by magnetic separation. Trizol reagent (Invitrogen/Life Technologies) was applied to isolated RNA from the complex. The miRNA expression was analyzed by qRT-PCR.

### Co-immunoprecipitation (co-IP)

Co-immunoprecipitation (co-IP) using PANC-1 cell lysate and antibody of SRSF1 (Santa Cruz, sc-33,652), EIF3B (Santa Cruz, sc-137,214), TIA1 (Santa Cruz, sc-166,247), GAPDH (ProMab, 20,035), and IgG (Santa Cruz sc-2025) was performed as described previously [33], and the protein complex was detected by western blot.

### Western blot analysis

Western blot was performed as we recently described [21, 26]. Primary antibodies raised against SRSF1 (Santa Cruz, sc-33,652), EIF3B (Santa Cruz, sc-137,214), TIA1 (Santa Cruz, sc-166,247), beta-actin (A5441), and Glyceraldehyde 3-phosphate dehydrogenase (GAPDH) (Santa Cruz, sc-47,724) were used for detection. Nuclear and cytoplasmic protein extraction was extracted following ROCKLAND Nuclear & Cytoplasmic Extract Protocol [34], and verified by Histone-H3 (CST, 4499S) and GAPDH (Santa Cruz, sc-47,724) detection. Antibodies used for exosome marker detection include: CD63, CD81 (Santa Cruz Bio Technology Inc., CA, USA), Flotillin-1 and Calnexin (Cell Signaling Technology, Inc., MA, USA).

### Quantitative real-time reverse transcription polymerase chain reaction (qRT-PCR)

qRT-PCR was performed as we described [21, 26] with specific primers: Cel-54 (5′-GCGCGCCCGTAATCTTCATAATCC-3′), miR-1246 (5′-GCGCGATGGATTTTTGGAGCAG-3′), miR-320c (5′-GCAAAAGCUGGGUUGAGAGGGU-3′), and miR-320d (5′-GCGAAAAGCUGGGUUGAGAGGA-3′).

### SRSF1 shRNA expression plasmid construction

Target specific oligonucleotides were designed using online tool RNAi Codex (Cold Spring Harbor Laboratory), and were synthesized (Integrated DNA Technologies) with the addition of overhangs according to the cutting site of BamH1 and EcoRI. The shRNA expression plasmid was constructed by annealing the oligonucleotides to pSIH-H1 vector following the user manual of pSIH-H1 shRNA system (SBI system Bioscience). The oligonucleotide sequences for shRNA of SRSF1, EIF3B or TIA1 are provided in Supplemental Table 1.

### Lentivirus transduction

Lentiviral particles were produced as previously described [35] using the shRNA expression plasmid and the 3rd generation packaging plasmids pMD2.G (Addgene plasmid #12259), pMDL/RREg/p (Addgene plasmid #12251), and pRSV-Rev (Addgene plasmid #12253). The packaging plasmids were co-transfected with the lentiviral expression vector into 293 T cells using the polyethyleneimine (Polysciences Inc.) to produce replication deficient lentivirus. After transfection, the supernatant was pooled and filtered with a 0.45 μm membrane and concentrated by ultracentrifugation to acquire lentivirus. Infection was performed by using lentivirus in the presence of 8 μg/ml polybrene (Sigma-Aldrich). Approximately 48 h post-infection cells were selected by treating with 10 μg/ml puromycin (InvivoGen, San Diego, CA).

### GFP-SRSF1 expression

The GFP-SRSF1 expression plasmid was a gift from Dr. Massimo Caputi [36]. DNA transfection was performed using Lipofectamine 3000 (Thermo Fisher Scientific) to PANC-1 cells and the expression of GFP-SRSF1 was verified by western blot.

### GST-SRSF1 protein purification

BL21 (Thermo-Fisher Scientific, C600003) competent cells transformed with pGEX6P-SRSF1 DNA (Addgene plasmid # 99020, [37] were cultured at 37 °C for 3.5 h, and after OD600 reached to 0.6–0.8, bacteria were treated with 0.1 mM isopropyl β-D-1-thiogalactopyranoside for 24 h at 16 °C. GST-tagged-SRSF1 was purified with Glutathione Sepharose beads (GE Health Care). The purity of the recombinant proteins was determined by SDS–PAGE with Coomassie blue staining.

### Electrophoretic mobility shift assay (EMSA)

IRD-800 labeled miR-1246 (0.1 μM) (Integrated DNA Technologies) was mixed with 4 μl of GST slurry, or GST-SRSF1 in binding buffer (Tris pH 8.0 40 mM, KCL 30 mM, MgCL2 1 mM, NP40 0.001%, DTT 1 mM, glycerol 5%) and incubated at room temperature for 40 min avoiding light. 5X loading buffer (KCL 60 mM, Tris PH 7.6 10 mM, glycerol 10%, xylene cyanol 0.01%, bromophenol blue 0.01%) was then added, and the complex was separated on a 4% native gel (40% polyacrylamide, 1 M Tris pH 7.5, 1 M glycine, 0.5 M EDTA, 10% APS, TEMED) at 120 voltage for 40 min. The signal was detected using the Li-Cor Odyssey 9120 Imaging system (LI-COR. Inc., USA).

### Design of decoy motif mimics

The decoy motif mimics were designed by permutation and combination of the identified motif sequences in the length of 24 nucleotides. The secondary structure of the designed sequences was analyzed in RNAfold WebServer (University of Vienna). Sequences without self-complementary were selected (Decoy mimics 1: 5′-UUGGACUAGGACUAGGAU-3′, Decoy mimics 2: 5′-AGGAAGGAAGGAAGGA-3′).

### Bioinformatics analysis

The miRNA motif analysis was performed using MEME Suite [38]. The protein profile analysis for the result of mass spectrometry was performed using DAVID Bioinformatics (ABCC at SAIC-Frederick, Inc). The RNA binding protein and miRNA sequence binding analysis was performed using the database of RNA-binding specificities (RBPDB) [39]. SRSF1 expression in cancer tissues was examined using ONCOMINE [40]. The correlation of gene expression with cancer patient survival was extracted from The Human Protein Atlas (SciLifeLab, Sweden) [41].

### Statistics

Statistical analyses were performed using GraphPad Prism software (GraphPad Software, Inc. La Jolla, CA, USA). The heatmap was made in RStudio (RStudio, Inc) with the ggplot2 package [42]. Student’s t-test was applied to determine significant differences among control and experimental groups.

## Results

### Identification of miR-1246 associated proteins

Because RBPs are involved in exosome miRNA sorting, we first sought to identify proteins that bind to miRNAs highly enriched in cancer exosomes. miR-1246, the most highly enriched miRNA in pancreatic cancer exosomes, was biotin-labeled and incubated with a cellular lysate from PANC-1 cells. The biotin-miR-1246 probe was captured with streptavidin-coated magnetic beads. Biotin labeled poly-A mimics were used as control. The miRNA-protein complexes were eluted and the proteins were analyzed by liquid chromatography/mass spectrometry in triplicate (Table 1). There were total of 593 proteins specifically pulled down by the miR-1246 probe. Interestingly, about half of the proteins that associate with miR-1246 are vesicle-associated proteins (Supplement Fig. 1A). Based on the intensity of detection, RNA binding property, and cancer relevance, we ranked the RBPs using “The Database for Annotation, Visualization and Integrated Discovery (DAVID)”. This resulted in ten candidate RBPs that complex with the miR-1246 sequence and are relevant to eukaryotic exosomes (Table 2). Among them, SRSF1 (also called SFRS1) was predicted to bind to the miR-1246 sequence (Supplement Fig. 2) by in silico analysis using the database of RNA-binding specificities (RBPDB) [39].

**Table 1:** Over view of the result of mass spectrometry

Experiments’ condition	Number of proteins detected
Poly A + PANC-1	575
Poly A + MDA-MB-231	609
miR-1246 + PANC-1	793
miR-1246 + MDA-MB-231	582

**Table 2:** miR-1246 RNA binding protein candidates obtained from the mass spectrometric analysis

Protein symbol	Protein full name
SRSF1	Serine/arginine-rich splicing factor 1
PARK7	Parkinson disease protein 7
EIF3B	Eukaryotic translation initiation factor 3 subunit B
THOC4	THO complex subunit 4 (Aly/REF export factor)
ACOC	Cytoplasmic aconitate hydratase
DDX5	Probable ATP-dependent RNA helicase DDX5
TIA1	T-cell-restricted intracellular antigen-1
IF5A1	Eukaryotic translation initiation factor 5A-1
EIF2A	Eukaryotic translation initiation factor 2A
IMDH2	Inosine-5′-monophosphate dehydrogenase 2

### Verification of SRSF1 binding to miR-1246

**Fig. 2:**
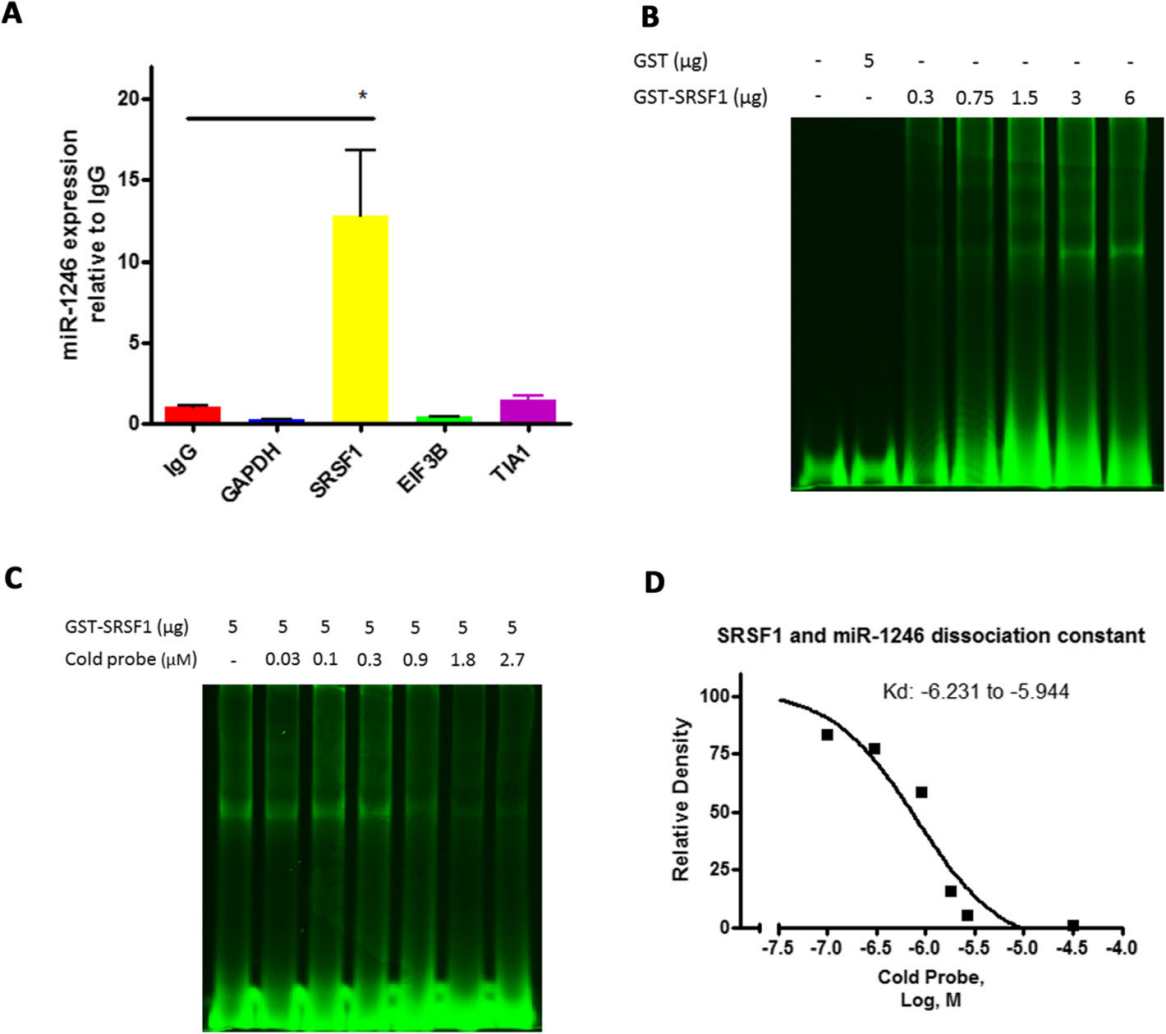
SRSF1 binds to miR-1246. **a** qRT-PCR detection of miR-1246 in IgG, GAPDH, SRSF1, EIF3B, and TIA1 immunoprecipitants of PANC-1 lysate (*n* = 3, *p < 0.001, student t-test). **b-c** EMSA detection of the SRSF1-miR-1246 complex (hot probe: IRD-800 labeled miR-1246 mimics; cold probe: miR-1246 mimics, *n* = 3). Direct binding of GST-SRSF1 and miR-1246 (**b**); and concentration-dependent competition between the cold and hot miR-1246 probe for binding to GST-SRSF1 (**c**). **d** Semi-quantification of SRSF1 and miR-1246 binding in ***C*** and calculated dissociation constant (*n* = 3)

RNA immunoprecipitation (RIP) was performed to verify the association of several identified RBPs with miR-1246, including SRSF1, EIF3B and TIA1. IgG and GAPDH antibody was used as controls for immunoprecipitation. As shown in Fig. 2a, miR-1246 expression is more than 12-fold higher in the SRSF1-precipitants, as compared to that of IgG precipitants, indicating a specific association of SRSF1 with miR-1246. miR-1246 expression was moderately increased in the TIA1-precipitants, and near IgG control levels in the EIF3B precipitants. Co-Immunoprecipitation (Co-IP) experiments were performed to verify the immunoprecipitation procedures (data not shown). To directly determine the binding of SRSF1 to the miR-1246 sequence, glutathione s-transferase (GST) conjugated human SRSF1 protein was expressed in BL21 competent *E. coli*, captured by glutathione sepharose beads, and eluted by glutathione. The purity of eluted GST-SRSF1 protein was shown by SDS-PAGE and Coomassie blue staining (Supplement Fig. 3).

The binding of GST-SRSF1 to a fluorescent-tagged miR-1246 probe was determined by RNA EMSA. As shown in Fig. 2b, binding of the labeled probe was specific to GST-SRSF1, but not GST, and increased with greater protein input. The specific binding of GST-SRSF1 to the miR-1246 probe was evident as the unlabeled miR-1246 probe effectively competed with the labeled miR-1246 probe in a concentration-dependent manner (Fig. 2c). The detected bands were semi- quantified and the K_d_ was calculated from the detected signals (Fig. 2d). These data confirmed the direct binding of SRSF1 to the miR-1246 sequence.

### Exosome miRNA enrichment by SRSF1 in cancer cells

**Fig. 3:**
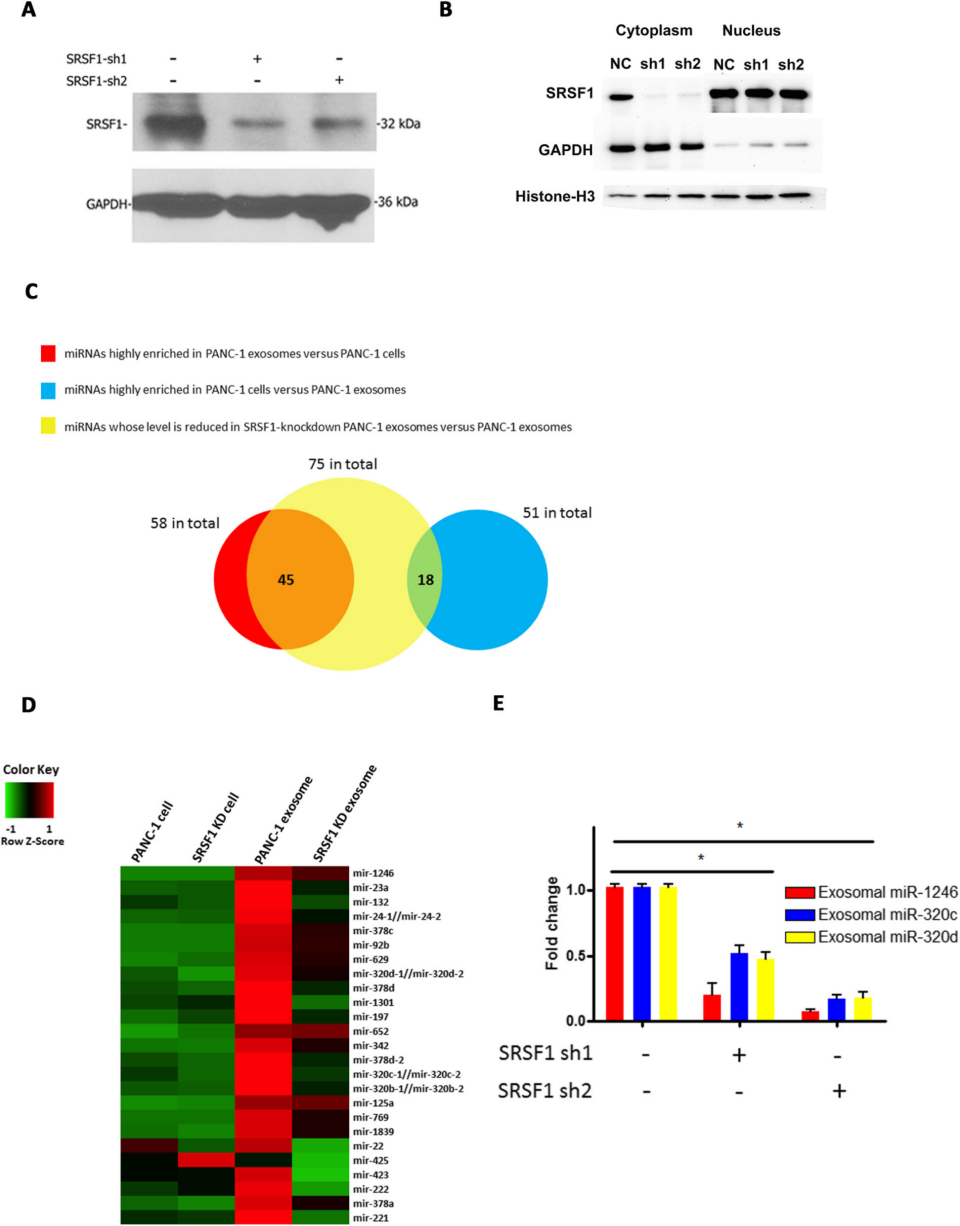
Cellular and exosome miRNA profiles after SRSF1 knockdown in PANC-1 cells. **a** Detection of SRSF1 knockdown by shRNAs in PANC-1 cells. **b** PANC-1 SRSF1 protein levels in nuclear and cytoplasmic fractions (NC: normal control). **c** Venn Diagram of overlap of miRNAs detected by next generation small RNA sequencing in SRSF1 knockdown and control PANC-1 cells and exosomes. **d** Heatmap showing the expression of top 25 exosome miRNAs in cells and exosomes after SRSF1 knockdown. **e** qRT-PCR analysis of miR-1246, miR-320c and miR-320d in exosomes derived from control and SRSF1 knockdown PANC-1 cells (*p < 0.001, Student’s t-test). Shown are representatives of three independent experiments (**a-e**)

Because SRSF1 is a key splicing factor that is essential to eukaryotic cells [31], a knockout model could not be established. Therefore, to determine whether SRSF1 miRNA binding activity is relevant to exosome miRNA enrichment, we established a lentivirus SRSF1 shRNA construct to knockdown SRSF1 expression in PANC-1 cells (Fig. 3a). Interestingly, though SRSF1 protein was detected both in the nucleus and cytoplasm, the knockdown was more pronounced in the cytoplasm (Fig. 3b). Knockdown of SRSF1 did not significantly alter the concentration and size distribution of the exosomes released by PANC-1 cells (Fig. 1b). Cellular and exosome RNA from control and SRSF1-shRNA cells were isolated and small RNA sequencing was performed. Among the 58 highly enriched PANC-1 exosome miRNAs, expression of 45 miRNAs (77.6%) was significantly down-regulated in exosomes derived from SRSF1-shRNA PANC-1 cells as compared to exosomes derived from control PANC-1 cells (Fig. 3c), strongly indicating the involvement of SRSF1 in exosome miRNA enrichment. A heatmap showing the expression of the top 25 miRNAs enriched in PANC-1 exosomes demonstrates the dramatic drop in expression levels of miRNAs in SRSF1-shRNA PANC-1 exosomes compared to PANC-1 exosomes (Fig. 3d). Notably, miR-1246 was the highest enriched exosome miRNA (data not shown) and its expression in exosomes was significantly reduced by SRSF1 knockdown (Fig. 3d). On the other hand, among 51 of the miRNAs less enriched in exosomes, only 18 (35.3%) were expressed at lower levels in exosomes derived from SRSF1 knockdown cells as compared to exosomes derived from wild type PANC-1 cells (Fig. 3c), suggesting that SRSF1 knockdown mainly affects exosome enriched miRNAs.

**Fig. 4:**
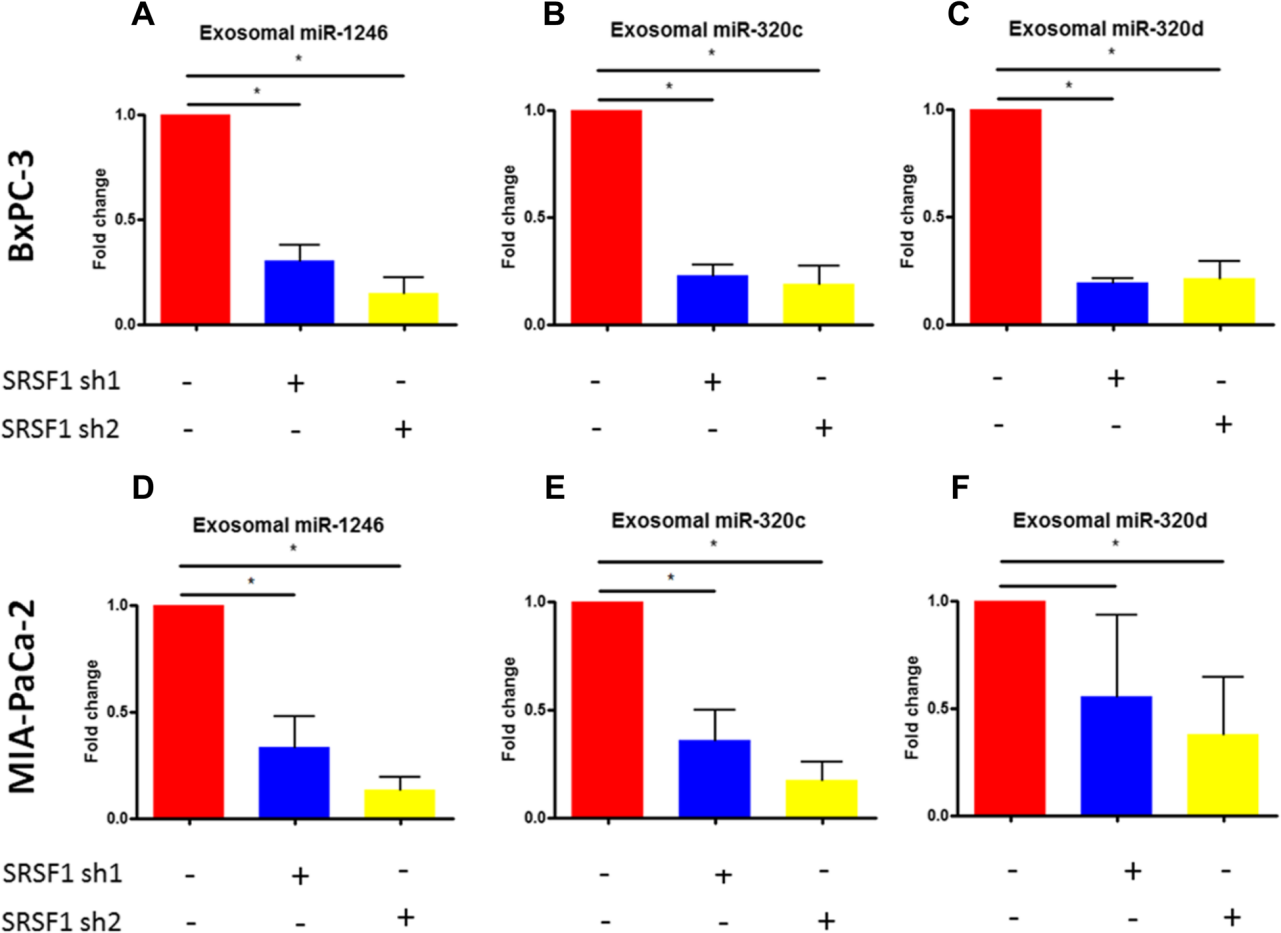
qRT-PCR analysis of miR-1246, miR-320c miR-320d expression in exosomes derived from SRSF1 knockdown BxPC-3 and MIAPaCa-2 cells. **a-c** qRT-PCR detection of miR-1246, miR-320c and miR-320d in exosomes derived from SRSF1 knockdown BxPC-3 cells (*n* = 3, *p < 0.001, Student t-test). **d-f** qRT-PCR detection of miR-1246, miR-320c and miR-320d in exosomes derived from SRSF1 knockdown MIAPaCa-2 cells (*n* = 3, *p < 0.001, Student’s t-test)

To further confirm the effect of SRSF1 knockdown on exosome miRNA enrichment, the expression levels of several representative miRNAs were quantified by qRT-PCR. SRSF1 knockdown in PANC-1 cells significantly reduced exosome levels of miR-1246, miR-320c and miR-320d, confirming the small RNA sequencing results (Fig. 3e). In contrast, knockdown of EIF3B or TIA1 did not reduce exosome miR-1246 expression, suggesting that these RBPs may not promote exosome miRNA enrichment (Supplement Fig. 4 and 5). Our observations were extended to two additional pancreatic cancer cell lines, MIAPaCa-2 and BxPC-3 (Fig. 4a-f). In addition, expression of let-7c, which is less enriched in exosomes, was unchanged in exosomes after SRSF1 knockdown (data not shown).

**Fig. 5:**
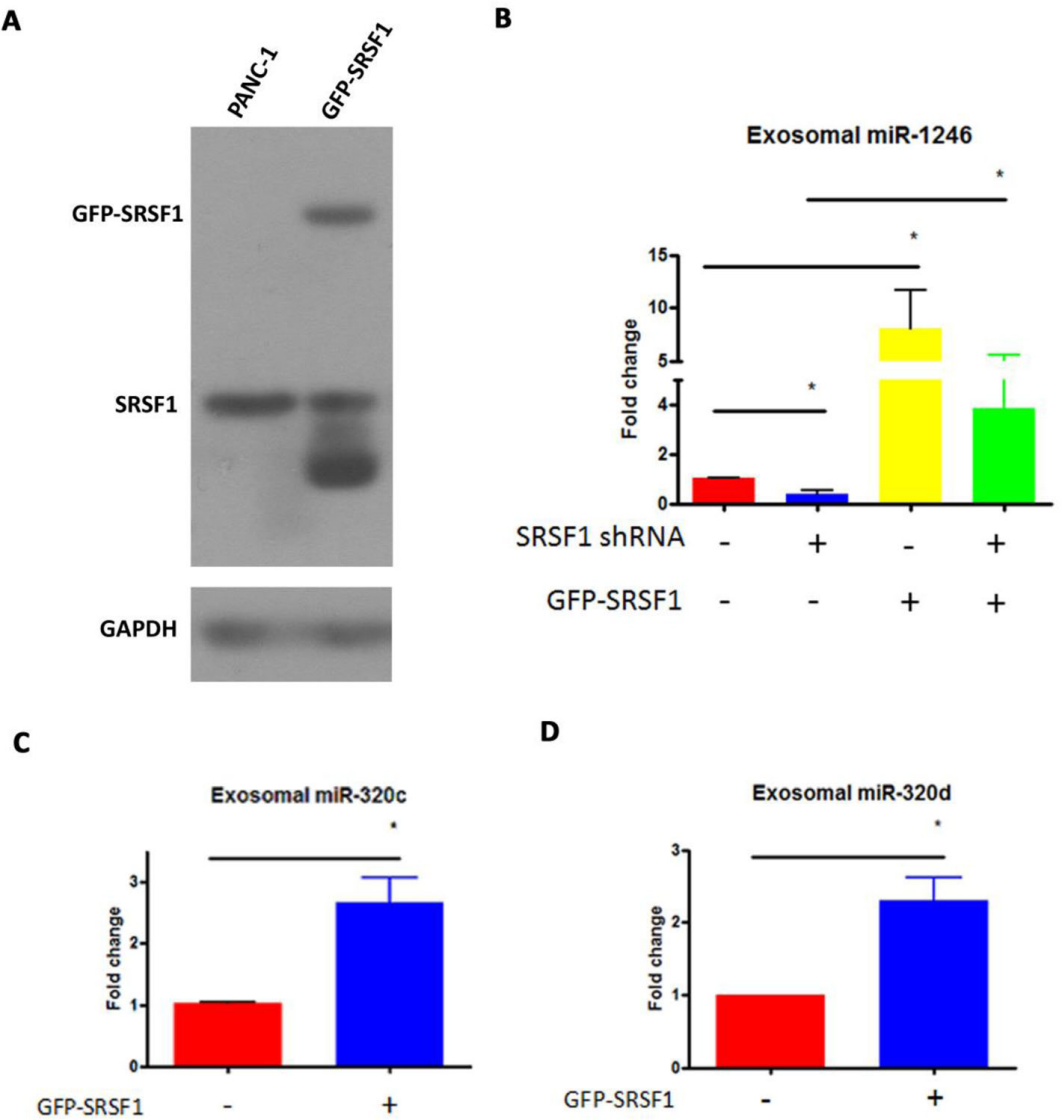
qRT-PCR analysis of exosome enriched miRNAs derived from SRSF1 overexpression PANC-1 cells. **a** Confirmation of GFP-SRSF1 overexpression in PANC-1 cells. **b** qRT-PCR detection of miR-1246 in exosomes derived from wild type and SRSF1 knockdown PANC-1 cells with GFP-SRSF1 overexpression. **c** qRT-PCR detection of miR-320c in exosomes derived from GFP-SRSF1 overexpression PANC-1 cells. **d** qRT-PCR detection of miR-320d in exosomes derived from GFP-SRSF1 overexpression PANC-1 cells. *p < 0.001, Student’s t-test, *n* = 3 for (**b-d**)

To verify the involvement of SRSF1 in exosome miRNA enrichment in cancer cells, we also exogenously overexpressed SRSF1 in PANC-1 cells. A GFP-SRSF1 expression plasmid was introduced into PANC-1 cells, and SRSF1 over-expression was confirmed by western blot (Fig. 5a). Expression of miR-1246, miR-320c, and miR-320d in the exosomes derived from GFP-SRSF1 PANC-1 cells was analyzed by qRT-PCR (Fig. 5b-c). As shown in Fig. 5b, over-expression of GFP-SRSF1 increased exosome expression of miR-1246 and rescued miR-1246 levels in exosomes derived from SRSF1-shRNA cells. Levels of miR-320c and miR-320d were also increased in exosomes derived from the GFP-SRSF1 cells, further supporting the involvement of SRSF1 in exosome miRNA enrichment (Fig. 5c-d).

### Identification of RNA sequence motifs involved in exosome miRNA enrichment

**Fig. 6:**
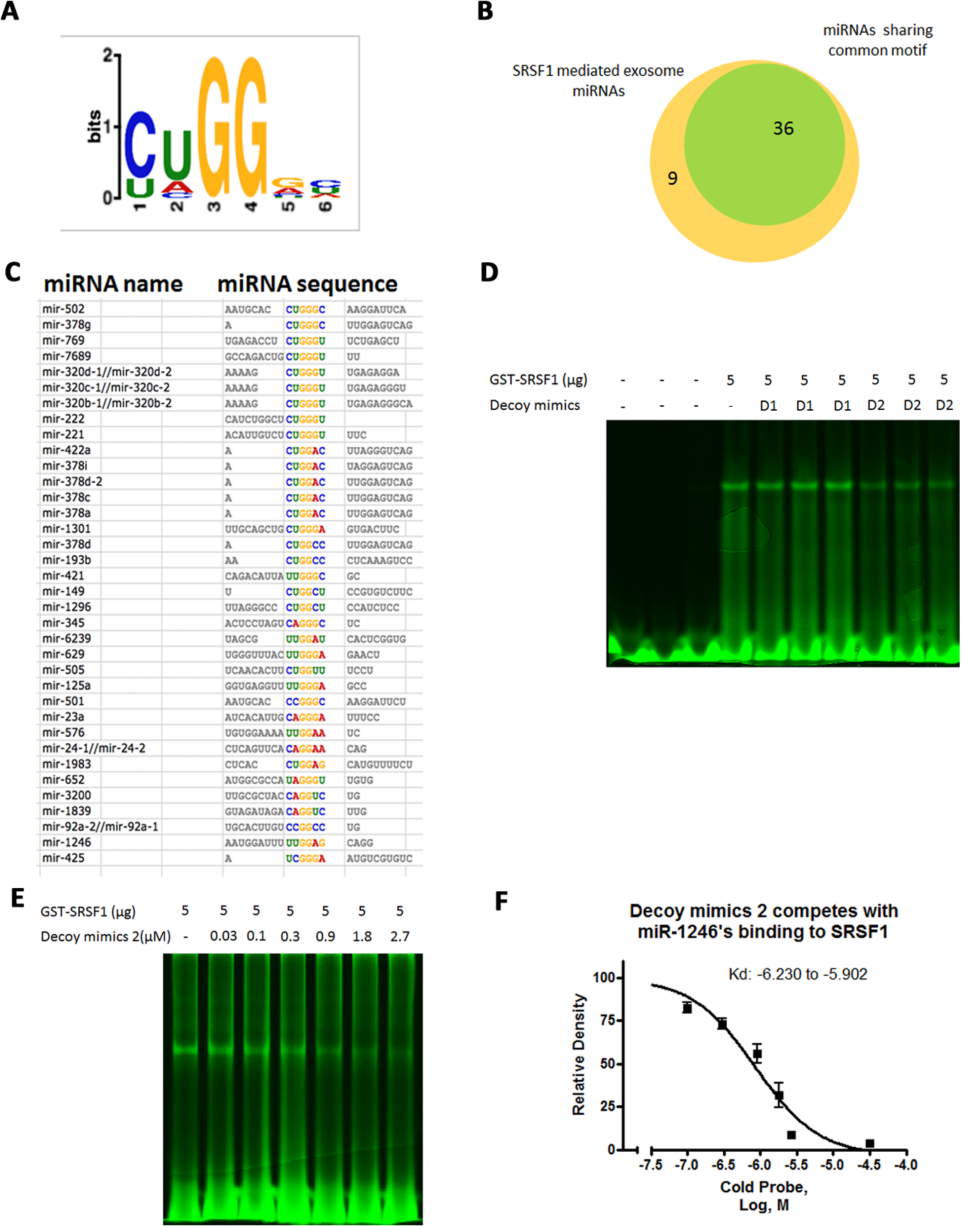
SRSF1-associated exosome miRNA sequence motif analysis. **a** The motif commonly shared among SRSF1-associated exosome miRNAs. **b** Venn diagram showing the number of SRSF1-associated exosome miRNAs that share the motif. **c** List of miRNAs sharing the common motif. **d** EMSA analysis demonstrating the inhibition of GST-SRSF1 binding to miR-1246 by RNA decoys (D1: decoy 1, 5′-UUGGACUAGGACUAGGAU-3′; D2: decoy 2, 5′-AGGAAGGAAGGAAGGA-3′). **e** Concentration-dependent inhibition of GST-SRSF1 binding to miR-1246 by D2. **f** Semi-quantification of the detected bands in Fig. 5e and the calculated dissociation constant

According to the RBPDB, SRSF1 binds specifically to a motif present in the miR-1246 sequence (Supplement Fig. 2). To understand the contribution of specific RNA motifs involved in exosome miRNA enrichment, we applied an unbiased approach to identify the RNA motifs that contribute to exosome miRNA enrichment. For this purpose, we analyzed the RNA sequences of the miRNAs highly enriched in cancer exosomes and regulated by SRSF1, using the bioinformatics tool MEME Suite [38]. A 6-bp length motif was found to be shared in 36 of the 45 exosome enriched miRNAs, including miR-1246 (Fig. 6 A-C). To test whether the binding of SRSF1 to miR-1246 depends on this motif, two decoy mimics were designed according to the shared motif sequences and their secondary structure (determined with the RNAfold WebServer, http://rna.tbi.univie.ac.at/cgi-bin/RNAWebSuite/RNAfold.cgi). The binding of the decoy mimics to SRSF1 protein was determined by RNA EMSA analysis. Addition of decoy motif #1 did not alter the binding of SRSF1 to the miR-1246 probe (Fig. 6d), whereas decoy motif #2 competed with miR-1246 binding to SRSF1 in a concentration-dependent manner (Fig. 6d-f), indicating that SRSF1 directly interacts with this sequence motif.

## Discussion

The role of exosome miRNA signaling in promoting cancer progression has been intensely investigated and well recognized in recent years [43, 44]. The higher enrichment of certain miRNAs in cancer exosomes [13, 19–21] indicates that exosome miRNA encapsulation is an active cellular process that initiates exosome miRNA signaling in the tumor microenvironment. However, the specific cellular process responsible for selective exosome miRNA enrichment has not been well established in eukaryotic cells. The most significant finding from the present study is that we have identified SRSF1 as a mediator of exosome miRNA enrichment in pancreatic cancer cells. A specific miRNA sequence motif was also identified that may be involved in the exosome miRNA enrichment process. These findings provide new insight into how miRNAs are enriched in cancer cell exosomes to initiate exosome-mediated miRNA signaling.

We recently reported that exosome miR-1246, the most highly enriched miRNA in pancreatic cancer cell-derived exosomes [21], is derived from RNU2–1, a small nuclear RNA important for mRNA splicing [26]. Along this line of our research, we sought to determine how this miRNA is enriched in cancer exosomes using our established model systems. In the present study, we have provided several lines of evidence demonstrating that SRSF1, a vital splicing factor [45] and established oncoprotein [46], is significantly involved in exosome miRNA enrichment in pancreatic cancer cells. The first line of evidence indicating SRSF1 involvement in exosome miRNA enrichment was obtained from the biotin-labeled miR-1246 pull-down experiment, followed by proteomic analysis. Among the RBPs identified, several were selected based on their detection intensity, relevance to extracellular vesicles, and reported connections to human cancer [40], including SRSF1, EIF3B, and TIA1. Of note, SRSF1 was the only RBP among them that was also predicted by the RBPDB to bind to a motif in the miR-1246 sequence. Furthermore, the direct binding of SRSF1 to the miR-1246 sequence was verified by RIP and RNA EMSA analysis, strongly indicating the physical interaction of SRSF1, and not EIF3B or TIA1, with the miR-1246 sequence. The most convincing evidence demonstrating the involvement of SRSF1 in cancer exosome miRNA enrichment was the observation that knockdown of SRSF1 significantly reduces exosome miRNA enrichment for a majority of the selectively enriched exosome miRNAs, without altering the expression levels of less enriched exosome miRNAs. These results were based on small RNA sequencing and confirmed by RT-PCR analysis. The observations were also extended to additional human pancreatic cancer cell lines, including MIAPaCa-2 and BxPC-3.

SRSF1 was initially identified as a splicing factor in eukaryotic cells [45], but SRSF1 was later revealed to shuttle between the nucleus and cytoplasm [47] to regulate RNA metabolism, miRNA procession [48] and other cellular events independent of the mRNA splicing process [31]. Importantly, SRSF1 is over-expressed in different cancer types and is considered a potent oncogene [46, 49]. Moreover, SRSF1 over expression in different types of cancer is associated with worse prognosis (Supplement Fig. 6). While the full spectrum of SRSF1 function remains to be determined, our results reveal that SRSF1 binds to specific miRNAs and is significantly involved in exosome miRNA enrichment in cancer cells. This function is likely independent of the splicing process as the reduced expression of the detected exosome miRNAs after SRSF1 knockdown is greater than their expression change in the cells. Because exosome miRNA signaling contributes to tumor development through intercellular communication in the tumor microenvironment [6, 50, 51], the involvement of SRSF1 in exosome miRNA signaling initiation likely represents a part of its oncogenic action, which may lead to new therapeutic strategies to intervene with exosome miRNA signaling in cancer. Several RBPs have been previously identified as mediators of exosome miRNA sorting in various model systems, including major vault protein in colon cancer cells [23], hnRNPA2B1 [29] in T cells, and YBX1 in HEK293T cells [30]. The identification of SRSF1 involvement in exosome miRNA enrichment in pancreatic cancer cells further supports the notion that the cellular exosome miRNA sorting process in eukaryotic cells may differ among different cell types.

We have also identified a miRNA motif commonly shared by the SRSF1-associated exosome miRNAs using the MEME Suite program (meme-suite.org). This motif was specifically bound by SRSF1 as evidenced by our RNA EMSA analysis. A similar motif, albeit slightly shorter, was identified in our recent report that describes exosome miR-1246 enrichment in pancreatic cancer cells [26]. Our results reinforce the concept that specific miRNA motifs are involved in exosome miRNA sorting [29]. The fact that a decoy motif was able to compete with the miR-1246 probe for binding to SRSF1 indicates a possibility that decoy motifs can be applied to alter exosome miRNA enrichment or exosome miRNA signaling in cancer cells. This assumption merits further investigation.

## Conclusions

In summary, we have demonstrated that SRSF1 binds to a specific motif in the miR-1246 sequence and is significantly involved in selective exosome miRNA enrichment in cancer cells. These findings reveal new insights into our understanding of the cellular process that initiates exosome miRNA signaling in cancer cells and may lead to the development of new therapeutic strategies against cancer.

## Supplementary information

**Additional file 1: Supplementary Table 1.** shRNA oligonucleotide sequences. **Supplementary Figure 1.** **Gene ontology analysis of miR-1246 associated proteins.** The enriched **A**. cellular component and **B.** molecular function categories of the 593 proteins found to associate with miR-1246. **Supplementary Figure 2.** Results of the RNA-binding specificities (RBPDB) database analysis of proteins predicted to bind to the mature miR-1246 sequence. **Supplement Figure 3.** **SDS-PAGE of purified GST-SRSF1.** A total of 5 μl of lysate or elute was loaded per lane. The gel was stained with Coomassie blue dye. **Supplementary Figure 4.** **Exosomal miR-1246 levels in EIF3B knockdown PANC-1 cells. A.** EIF3B knockdown verified by western blot. **B.** Exosomal miR-1246 in EIF3B knockdown PANC-1 cells. **Supplementary Figure 5.** **Exosomal miR-1246 in TIA1 knockdown PANC-1 cells. A**. TIA1 knockdown verified by western blot. **B.** Exosomal miR-1246 in TIA1 knockdown PANC-1 cells. **Supplementary Figure 6.** **Association of SRSF1’s expression level with cancer patient survival.** Data were from THE HUMAN PROTEIN ATLAS database: **A**. SRSF1 expression and survival years in pancreatic cancer patients. **B**. SRSF1 expression and survival years in liver cancer patients. **C.** SRSF1 expression and survival years in renal cancer patients.

## Data Availability

The data generated or analyzed during this study are included in this manuscript and its supplementary files or are available from the corresponding author on reasonable request.

## Abbreviations

ATCC: American type culture collection
EIF3B: Eukaryotic translation initiation factor 3 subunit B
Co-IP: Co-immunoprecipitation
EMSA: Electrophoretic mobility shift assay
GAPDH: Glyceraldehyde 3-phosphate dehydrogenase
miRNA: MicroRNA
qRT-PCR: Quantitative reverse transcription PCR
RBP: RNA binding protein
RIP: RNA immunoprecipitation
SRSF1: Serine and arginine rich splicing factor 1
TIA1: T cell-restricted intracellular antigen 1
