# Supplementary material for: SRSF1 regulates exosome microRNA enrichment in human cancer cells
Source: Cell Commun Signal. 2020 Aug 20;18:130. doi: 10.1186/s12964-020-00615-9 (PMC7439691; doi:10.1186/s12964-020-00615-9)
Supplement: Supplementary file 2 — Additional file 1: Supplementary Table 1. shRNA oligonucleotide sequences. Supplementary Figure 1. Gene ontology analysis of miR-1246 associated proteins. The enriched A. cellular component and B. molecular function categories of the 593 proteins found to associate with miR-1246. Supplementary Figure 2. Results of the RNA-binding specificities (RBPDB) database analysis of proteins predicted to bind to the mature miR-1246 sequence. Supplement Figure 3. SDS-PAGE of purified GST-SRSF1. A total of 5 μl of lysate or elute was loaded per lane. The gel was stained with Coomassie blue dye. Supplementary Figure 4. Exosomal miR-1246 levels in EIF3B knockdown PANC-1 cells. A. EIF3B knockdown verified by western blot. B. Exosomal miR-1246 in EIF3B knockdown PANC-1 cells. Supplementary Figure 5. Exosomal miR-1246 in TIA1 knockdown PANC-1 cells. A. TIA1 knockdown verified by western blot. B. Exosomal miR-1246 in TIA1 knockdown PANC-1 cells. Supplementary Figure 6. Association of SRSF1’s expression level with cancer patient survival. Data were from THE HUMAN PROTEIN ATLAS database: A. SRSF1 expression and survival years in pancreatic cancer patients. B. SRSF1 expression and survival years in liver cancer patients. C. SRSF1 expression and survival years in renal cancer patients. [file 12964_2020_615_MOESM1_ESM.docx]

**Supplementary Table 1:** shRNA oligonucleotide sequences

| Oligos Name | Sequence (5’ to 3’) |
| --- | --- |
| SFRS1 sh1 F | AGCGCGCAGAGGATCACCACGCTATTTAGTGAAGCCACAGATGTAAATAGCGTGGTGATCCTCTGCT |
| SFRS1 sh1 R | GGCAAGCAGAGGATCACCACGCTATTTACATCTGTGGCTTCACTAAATAGCGTGGTGATCCTCTGCG |
| SFRS1 sh2 F | AGCGCGGGCCCAGAAGTCCAAGTTATTAGTGAAGCCACAGATGTAATAACTTGGACTTCTGGGCCCA |
| SFRS1 sh2 R | GGCATGGGCCCAGAAGTCCAAGTTATTACATCTGTGGCTTCACTAATAACTTGGACTTCTGGGCCCG |
| TIA1 sh1 F | AGCGGAGTTGACATGAGATTTATATAGTGAAGCCACAGATGTATATAAATCTCATGTCAACTC |
| TIA1 sh1 R | GGCAGAGTTGACATGAGATTTATATACATCTGTGGCTTCACTATATAAATCTCATGTCAACTC |
| TIA1 sh2 F | AGCGGCCGTTGTTTACTTAAAGATTAGTGAAGCCACAGATGTAATCTTTAAGTAAACAACGGC |
| TIA1 sh2 R | GGCAGCCGTTGTTTACTTAAAGATTACATCTGTGGCTTCACTAATCTTTAAGTAAACAACGGC |
| EIF3b sh1 F | AGCGAGGGTCAACCTCTTTACGGATTTAGTGAAGCCACAGATGTAAATCCGTAAAGAGGTTGACCCG |
| EIF3b sh1 R | GGCACGGGTCAACCTCTTTACGGATTTACATCTGTGGCTTCACTAAATCCGTAAAGAGGTTGACCCT |
| EIF3b sh2 F | AGCGAGGAGAGAAATTCAAGCAAATTTAGTGAAGCCACAGATGTAAATTTGCTTGAATTTCTCTCCC |
| EIF3b sh2 R | GGCAGGGAGAGAAATTCAAGCAAATTTACATCTGTGGCTTCACTAAATTTGCTTGAATTTCTCTCCT |

Supplementary Figure 1

**A B**


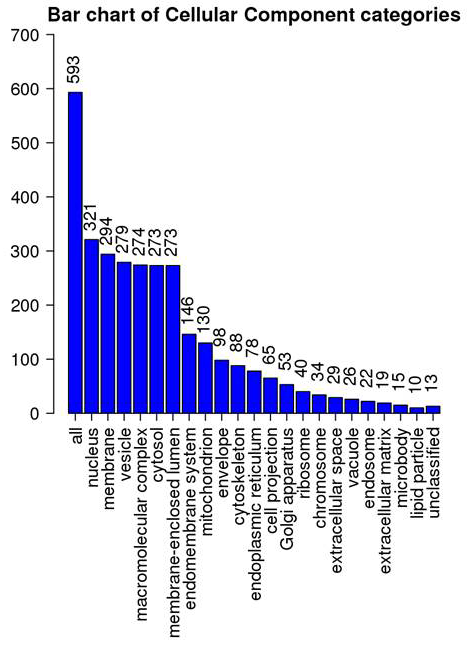

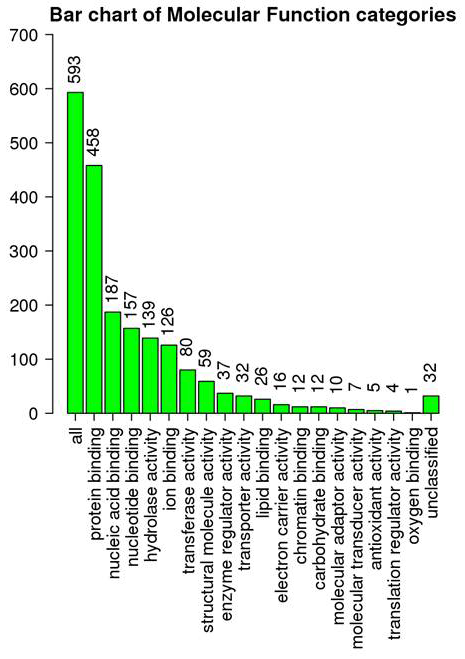


**Gene ontology analysis of miR-1246 associated proteins.** The enriched **A**. cellular component and **B.**  molecular function categories of the 593 proteins found to associate with miR-1246.

Supplementary Figure 2


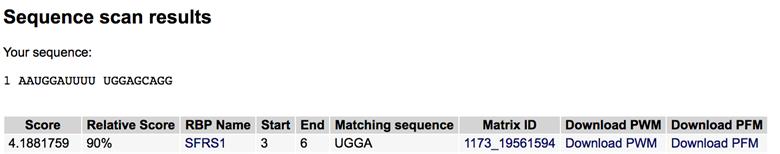


**Results of the RNA-binding specificities (RBPDB) database analysis of proteins predicted to bind to the mature miR-1246 sequence.**

Supplement Figure 3


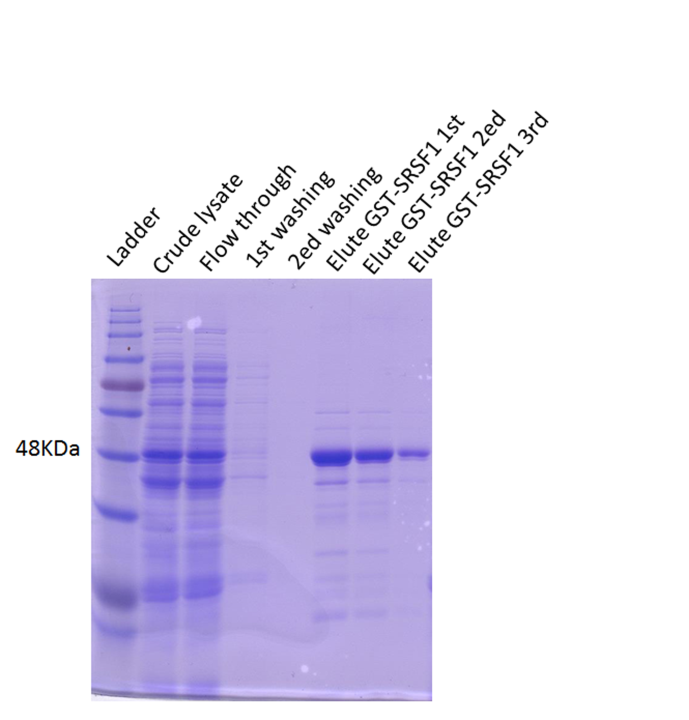


**SDS-PAGE of purified GST-SRSF1.** A total of 5μl of lysate or elute was loaded per lane. The gel was stained with Coomassie blue dye.

Supplementary Figure 4


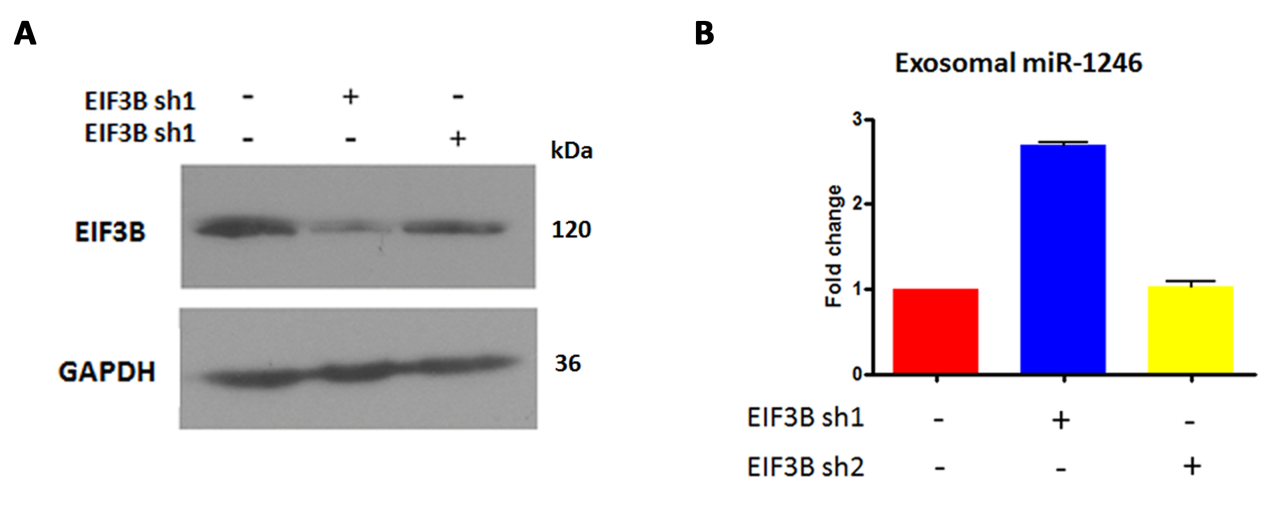


**Exosomal miR-1246 levels in EIF3B knockdown PANC-1 cells. A.** EIF3B knockdown verified by western blot. **B.** Exosomal miR-1246 in EIF3B knockdown PANC-1 cells.

Supplementary Figure 5


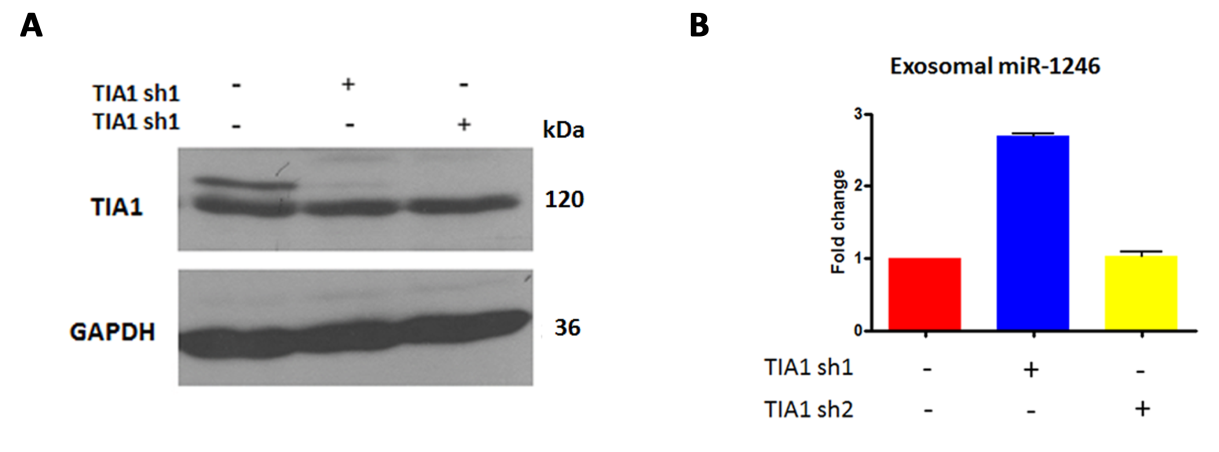


**Exosomal miR-1246 in TIA1 knockdown PANC-1 cells. A**. TIA1 knockdown verified by western blot. **B.** Exosomal miR-1246 in TIA1 knockdown PANC-1 cells.

Supplementary Figure 6


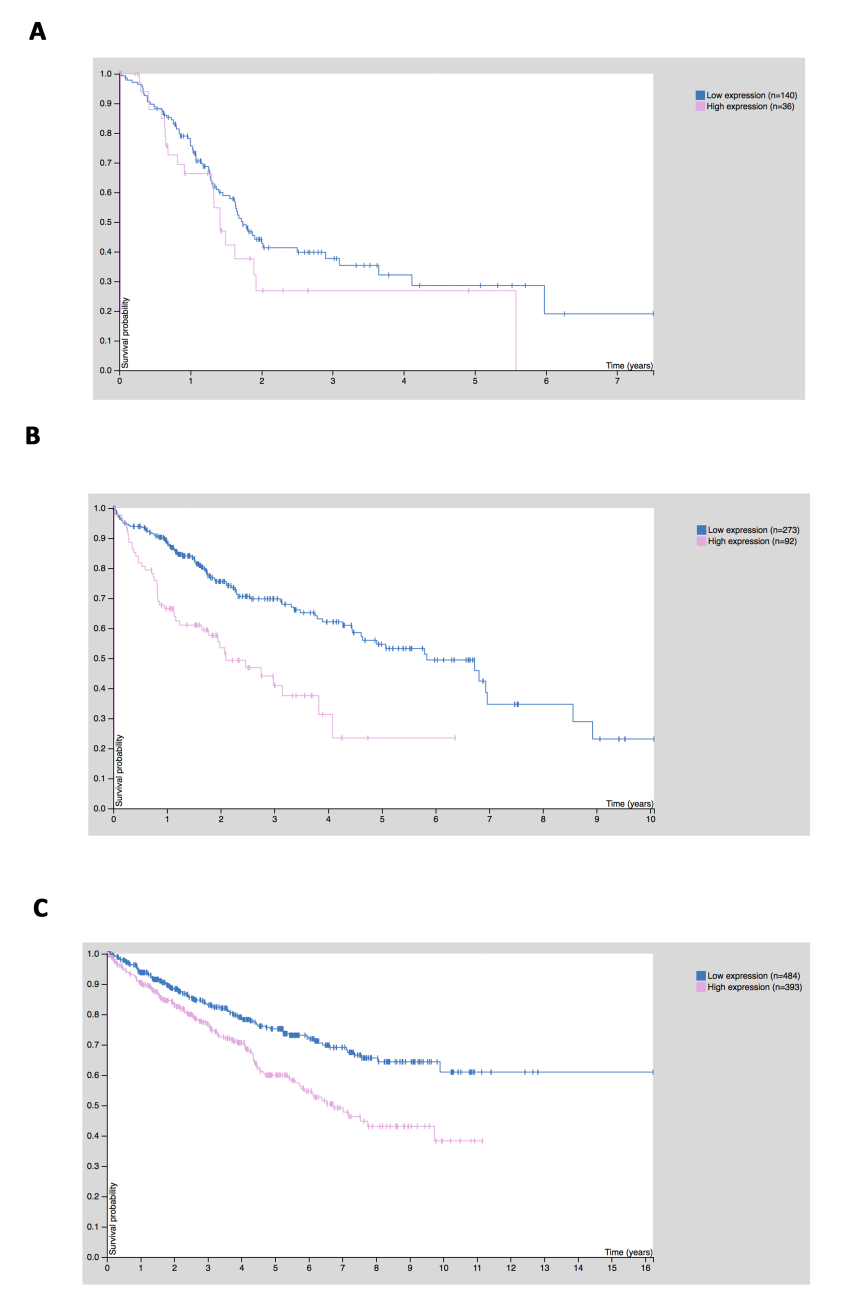


**Association of SRSF1’s expression level with cancer patient survival.** Data were from THE HUMAN PROTEIN ATLAS database**:** **A**. SRSF1 expression and survival years in pancreatic cancer patients. **B**. SRSF1 expression and survival years in liver cancer patients. **C.** SRSF1 expression and survival years in renal cancer patients.
